# Characterization of piroctone olamine for topical delivery to the skin

**DOI:** 10.1111/ics.12839

**Published:** 2023-03-27

**Authors:** Chun Fung Tang, Miguel Paz‐Alvarez, Paul D. A. Pudney, Majella E. Lane

**Affiliations:** ^1^ UCL School of Pharmacy London UK; ^2^ Unilever R&D Port Sunlight Bebington UK

**Keywords:** distribution coefficient, piroctone olamine, pre‐formulation, solubility, stability

## Abstract

**Objective:**

Dandruff and its more severe related condition, seborrheic dermatitis affects a high proportion of the population at some point in their life. Piroctone olamine, also known as Octopirox® (OPX) is the monoethanolamine salt of piroctone and is an antifungal agent widely used for the management of dandruff. The aim of the present work was to characterize the physicochemical properties of piroctone olamine and to conduct pre‐formulation studies for the development of novel topical formulations of this active.

**Methods:**

An HPLC method was developed and validated for the analysis of OPX. The melting point was determined using the DSC Q2000 (TA Instruments, USA). The distribution coefficient (log*D*_(O/PBS)_) and partition coefficient (log *P*_o/w_) was determined in phosphate‐buffered saline (PBS) AND deionized (DI) water using the shake flask method. All experiments were performed at room temperature. The solubility was determined experimentally by adding amount of active to a solvent. The samples were kept at 32° ± 1°C for 48 h in a water bath. The stability of the compound was determined in a range of solvents by preparing solutions of 1 mg mL^−1^ in the relevant solvents. These solutions were kept and stirred throughout the experiment at 32 ± 1°C, and aliquots were taken at 24, 48 and 96 h.

**Results:**

The HPLC method was developed successfully; however, samples at the lower end of the calibration curve showed lower degrees of precision and accuracy. Based on experiments with DSC, the melting point was observed at an onset temperature of 132.4°C. The Log*D* was determined to be 1.84. The compound had the highest solubility in methanol (278.4 mg mL^−1^) and propylene glycol (PG), with a value of 248.8 mg mL^−1^. The lowest solubility for OPX was in dimethyl isosorbide (9.9 mg mL^−1^), Labrafac™ (3.6 mg mL^−1^) and isostearyl isostearate (0.5 mg mL^−1^). Over the 4 days, OPX showed stability in ethanol and PG, while a notable decrease in OPX was observed in PBS and DI water at 32 ± 1°C.

**Conclusion:**

The physicochemical properties of OPX were characterized to find suitable excipients able to target the epidermis for topical delivery. Building on these findings, future work will focus on the development of novel topical formulation of OPX.

## Graphical abstract

Piroctone olamine, also known as Octopirox® (OPX) had the highest solubility in methanol and propylene glycol. To our knowledge, this has not been reported previously.

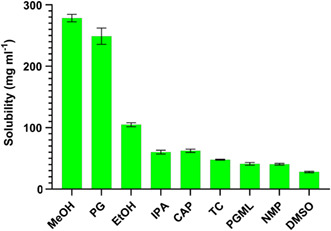

## Résumé

**Objectif:**

la production de pellicules et la maladie plus grave qui y est apparentée, la dermatite séborrhéique, touchent une grande partie des personnes à un moment donné de leur vie. La piroctone olamine, également connue sous le nom d’Octopirox® (OPX), est le sel de monoéthanolamine de la piroctone. Il s’agit d’un agent antifongique largement utilisé pour le traitement des pellicules. L’objectif de ce travail était de caractériser les propriétés physicochimiques de la piroctone olamine et de mener des études de préformulation pour le développement de nouvelles formulations topiques de ce principe actif.

**Méthodes:**

une méthode de chromatographie liquide à haute performance (CLHP) a été développée et validée pour l’analyse de l’OPX. Le point de fusion a été déterminé à l’aide du calorimètre à balayage différentiel (Differential Scanning Calorimetry, DSC) Q2000 (TA Instruments, États‐Unis). Le coefficient de distribution (logD_(Octanol/PBS)_) et le coefficient de partage (log P_octanol/eau_, ou log P_oe_) ont été déterminés dans le tampon phosphate salin (phosphate buffered saline, PBS) et dans l’eau désionisée (deionised, DI) à l’aide de la méthode par agitation en flacon. Toutes les expériences ont été réalisées à température ambiante. La solubilité a été déterminée de manière expérimentale. Une certaine quantité du principe actif a été ajoutée au solvant. Les échantillons ont été conservés à une température de 32 °C ± 1 °C pendant 48 h dans un bain‐marie. La stabilité du composé a été déterminée à l’aide d’une gamme de solvants. Des solutions de 1 mg mL^−1^ ont été préparées dans les solvants correspondants. Les solutions ont été conservées et agitées tout au long de l’expérience à une température de 32°C ± 1°C. Des aliquotes ont été prélevées après 24, 48 et 96 h.

**Résultats:**

la méthode CLHP a été développée avec succès. Toutefois, les échantillons situés dans la partie inférieure de la courbe d’étalonnage ont montré des degrés inférieurs de précision et d’exactitude. Sur la base des expériences avec le DSC, le point de fusion a été observé à une température initiale de 132,4°C. Le LogD a été déterminé à 1,84. Le composé présentait la solubilité la plus élevée dans le méthanol (278,4 mg mL^−1^) et le propylène glycol (PG), avec une valeur de 248,8 mg mL^−1^. L’OPX présentait la solubilité la plus faible dans l’isosorbide de diméthyle (9,9 mg.mL^−1^), le Labrafac^TM^ (3,6 mg mL^−1^) et l’isostéarate d’isostéaryle (0,5 mg mL^−1^). Sur les 4 jours, l’OPX a montré une stabilité dans l’éthanol et le PG, tandis qu’il a diminué de manière notable dans le PBS et l’eau désionisée à une température de 32°C ± 1°C.

**Conclusion:**

les propriétés physicochimiques de l’OPX ont été caractérisées afin de trouver des excipients appropriés capables de cibler l’épiderme dans le cadre d’une administration topique. En s’appuyant sur ces résultats, les travaux futurs se concentreront sur le développement d’une nouvelle formulation topique de l’OPX.

## INTRODUCTION

Extending across an area of up to 2 m^2^ in adults, the skin is the largest organ of our body by mass [1]. Even though it had served for millennia as a delivery route, the skin was regarded as an impermeable barrier until early in the 20th century [2]. However, it was not until the mid‐1900s that an ointment was applied topically for a curative effect [3]. An ointment is possibly one of the simplest delivery systems for actives, investigated by Higuchi [4], whose seminal contributions led to what we know today as formulation science, the influence of the formulation on the delivery of an active ingredient.

Formulation is key to the efficacy of topical products for the skin [5]. While a petrolatum ointment may constitute an effective vehicle, creating an elegant, consumer‐friendly preparation should be the goal of the formulation scientist. Therefore, several steps are required for the development of a final product. The right balance needs to be found between the target effect and a pleasant feel upon application. It is also the role of the formulation scientist to provide safe products to the consumer through extensive testing before release into the market. To find the right formulation to meet the aforementioned premises, a one‐size‐fits‐all approach is not appropriate, as the formulation needs to marry with the purpose and the characteristics of the active ingredient. Physicochemical properties such as partition coefficient (Log *P*_o/w_), melting point or pK_a_ have been traditionally identified as good predictors of skin delivery [6, 7]. As these parameters are not always known or reported; they must be characterized experimentally for certain actives.

**FIGURE 1:**
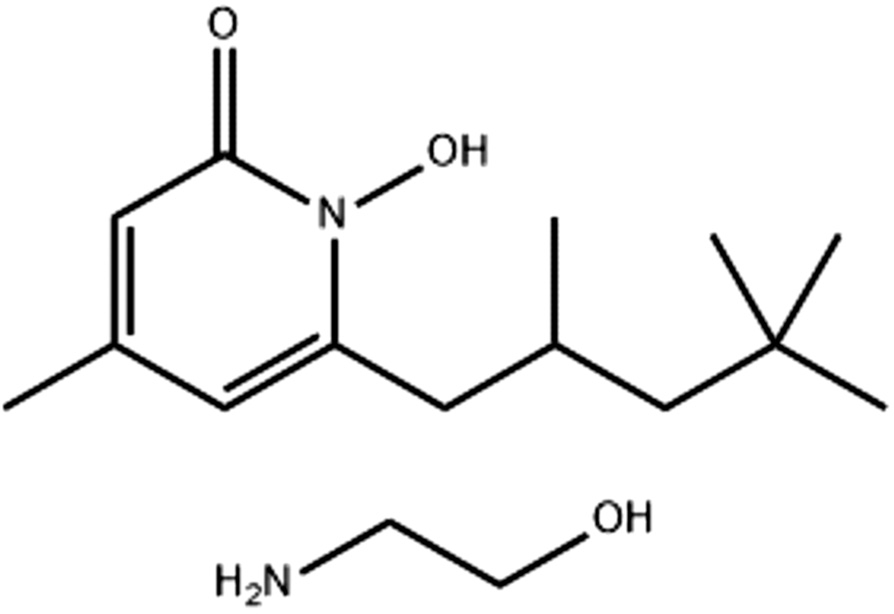
Chemical structure of piroctone olamine.

Piroctone olamine also known as Octopirox® (OPX), shown in Figure 1, is currently used in commercial formulations for the treatment of dandruff. Despite the demonstrated clinical efficacy of this compound, there is very limited information regarding the physicochemical properties of OPX in the literature. In order to design effective formulations, the active ingredients, as well as candidate excipients and solvents, need to be fully characterized before conducting further in vitro studies. Thus, the aims of the present work were to (i) develop and validate methods for the analysis of OPX, (ii) determine the physicochemical properties of OPX and (iii) identify appropriate solvents/excipients for the topical formulation of OPX.

## MATERIALS AND METHODS

### Materials

OPX was donated by Unilever, Bedford, UK. Propylene glycol (PG), 2‐ethylhexyl salicylate (OSal), *n*‐methyl‐2 pyrrolidone (NMP), dl‐dithiothreitol (DTT), ethylene diamine tetraacetic acid (EDTA) and *n*‐octanol were purchased from Sigma Aldrich, UK. Transcutol® P (TC), propylene glycol caprylate (CAP), propylene glycol monolaurate (PGML) and caprylic/capric triglycerides (LAB) were kindly gifted from Gattefossé, (St. Priest, France). Dimethyl isosorbide (DMI) and isostearyl isostearate (ISIS) were provided as free samples by Croda (Goole, UK). HPLC grade DI water, acetonitrile (ACN) and methanol, isopropanol (IPA), absolute ethanol (EtOH), sodium di‐hydrogen orthophosphate, di‐sodium hydrogen orthophosphate, trifluoroacetic acid and dimethyl sulfoxide (DMSO) were purchased from Fisher Scientific, UK.

### Melting point calculation

A Tzero™ press (TA instruments, USA) was used to seal the pan containing OPX hermetically with its corresponding lid (TA instruments, USA) and an empty pan sealed with its lid was used as the reference. The samples were then heated from −20 to 140°C at 10°C min^−1^ under N_2_ gas (50 mL min^−1^) in a differential scanning calorimeter Q2000, (TA Instruments, USA). Data analysis was performed with TA Universal Analysis software.

### HPLC/LC–MS method for OPX

HPLC analysis was performed using a 1100 series HPLC (Agilent, USA) and a reverse phase C_8_ column (Luna 5 μm, pore size 100 Å, 150 × 4.6 mm; Phenomenex, UK). The mobile phase was a gradient mixture of 0.1% v/v trifluoroacetic acid (TFA) in water and ACN as described in Table 1. The column temperature, mobile phase flow rate and injection volume were set at 32°C, 1 mL min^−1^ and 20 μL, respectively. The optimum detection wavelength was determined by UV absorption and set to 305 nm. Validation of the method was conducted according to ICH guidelines [8].

**TABLE 1:** Mobile phase gradient for the detection of OPX.

Time (min)	0.1% TFA in water (%)	ACN (%)
0.0	35	65
4.0	20	80
6.0	3	97
6.5	35	65

As OPX is formed of two ions, mass spectrometry (MS) was used to identify which species were being detected with HPLC‐UV. Therefore, an LC–MS method was developed in a G6460 Triple Quadrupole (QQQ) LC–MS instrument (Agilent, UK). The same parameters in terms of column, mobile phase, injection volume, column temperature and flow rate were used as described for HPLC. The QQQ was set to positive polarity and the acquisition range was limited to *m*/*z* values of 100–800 in an MS/MS scan for the samples eluting from 2 to 8 min. Analytes reached the MS through electrospray ionization using Agilent Jet Stream technology with super‐heated nitrogen to improve ion generation and desolvation. The gas temperature was set to 300°C at a flow of 5 L min^−1^ and the nebuliser was kept at 45 psi. Quantitative data analysis was performed using Agilent MassHunter software (Agilent, UK) following total ion chromatography, which includes all mass spectral peaks for each scan, including background noise and sample components.

### Distribution and partition coefficient determination (log *D*_o/PBS_, log *P*_o/w_)

The distribution (log *D*_O/PBS_) and partition coefficient (log *P*_O/W_) for OPX was determined using the shake flask method [9]. The log *D*_O/PBS_ was determined in PBS and log P_o/w_ was determined in DI water. *N*‐octanol was used as the organic phase and all experiments were performed at room temperature (23 ± 2°C). The amount of active in each phase in the different ratios was determined using HPLC to calculate the log *D*_O/PBS_ using Equation (1) where *C*_*n*‐octanol_ is the concentration in the organic phase and *C*_aqueous_ the concentration in the relevant aqueous phase:

(1) $$\;\log\;P_{O/W}=\log\;D_{O/\mathrm{PBS}}=\log\left(\frac{C_{n-\text{octanol}}}{C_{\text{aqueous}}}\right)$$

### Solubility and stability studies

The rationale underlying selection of solvents was the requirement to identify a solvent for extraction of active from the skin and to prepare solvents or solvent systems with a minimum concentration of 1% of the active. An initial amount of active was added to 0.5 mL of solvent. Small flat bottom tubes with plastic caps and PTFE‐coated stirring bars were used. The tubes were submerged in a JB Nova thermostatically controlled water bath (Grant, UK) equipped with an HP 15 stirring system (Variomag®, USA) at 32° ± 1°C for 48 h. More active was added if needed until saturation (visible crystals) and the caps were covered with Parafilm® (Bemis Company Inc., USA). The centrifuge was preheated at 32°C while the saturated solutions were placed in microcentrifuge tubes inside the water bath. These were centrifuged at 13 200 rpm for 30 min at 32°C; then, the supernatant was then carefully withdrawn and suitably diluted to lie within the range of the calibration curve. Finally, the samples were analysed by HPLC. All measurements were conducted in triplicate.

The stability of OPX was investigated in vehicles that were selected for in vitro studies (*n* = 3). Stability in PBS and 5% (w/v) Brij® O20 PBS (Merck, Germany) was also studied to ensure the actives would be stable in the receptor compartment over the course of the experiments. Solutions of 1 mg mL^−1^ were prepared in the relevant solvents and placed in flat bottom screw top glass vials, closed with plastic caps and covered in Parafilm to avoid evaporation of the samples. Solutions were stirred throughout the experiment using PTFE‐covered magnetic stirrers. The vials were introduced in a SUB thermostatically controlled water bath equipped with an HP 14 stirring system at 32 ± 1°C. Aliquots were taken at 24, 48 and 96 h and diluted accordingly to fit the calibration curve. The HPLC analysis was performed following the method described earlier.

### Data analysis

Data were plotted using Microsoft® Excel (Microsoft, USA) and GraphPad Prism® (Graphpad software, USA). The statistical analysis was carried out using GraphPad Prism®. The results were assessed for normality using the Shapiro–Wilk test, and the homogeneity of variance was assessed using Levene's test one‐way ANOVA and Independent samples t‐test were performed where appropriate. Multiple comparison Tukey's HSD post hoc test was used after ANOVA analysis to perform pairwise analysis. For non‐normally distributed data or where variances were not equal, the Kruskal–Wallis and Mann–Whitney *U* tests and independent sample t‐test were used, respectively. A *p*‐value lower than 0.05 (*p* < 0.05) was considered statistically significant.

## RESULTS AND DISCUSSION

### Melting point

**FIGURE 2:**
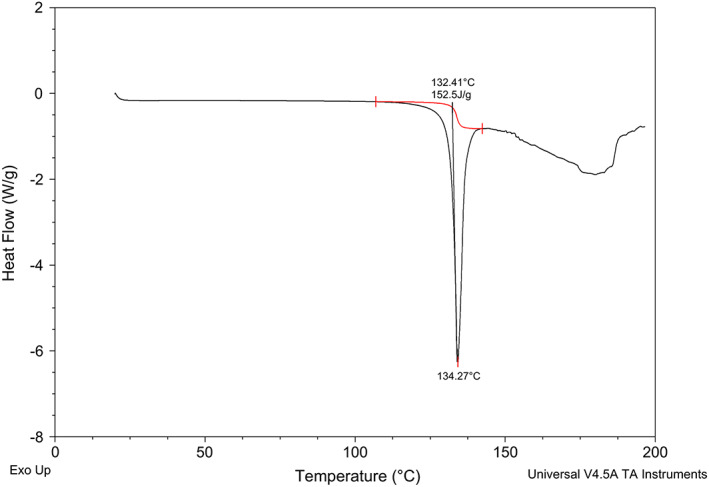
DSC thermogram of OPX.

From the DSC thermogram (Figure 2), one main endothermic event was observed at an onset temperature of 132.4°C, which corresponds to the melting point of OPX and provides information about the crystal state of the solid. There is no reported reference about crystal polymorphism, but the same melting temperature was reported by ToxServices [10] and no further endotherms suggest that this would be the most stable polymorph. As temperatures rise above 133°C, degradation of OPX is observed in an erratic and broad endotherm.

Melting point has been reported to show some degree of correlation with the permeability coefficient (*K*_p_) for a range of chemicals [11]. For the majority of commercialized topical actives, melting points lower than 250°C seem to be indicative of favourable properties for permeation [1].

### OPX HPLC method validation

Table 2 shows the HPLC method validation for OPX. Samples within the lower end of the calibration curve showed lower degrees of precision and accuracy, which could be due to their vicinity to the LOD of 0.5 μg mL^−1^. OPX was dissolved in methanol and linearity was confirmed from 0.5 to 50 μg mL^−1^.

**TABLE 2:** HPLC method validation results for quantitative analysis of OPX. Mean ± SD, precision expressed in RSD%.

Linearity	>0.999		
Specificity	No other interferences		
Analytical limits			
LOD (μg mL^−1^)	0.50		
LOQ (μg mL^−1^)	1.51		
System suitability			
Retention time	4.4 ± 0.2		
Symmetry	0.87 ± 0.05		
Accuracy			
Concentration (μg mL^−1^)	100	15	1
Recovery (%)	99.4 ± 0.3	99.7 ± 1.2	109.7 ± 1.9
Precision			
Intraday (RSD%)	0.33	1.17	1.71
Interday (RSD%)	0.98	8.32	9.6

OPX is formed of an acidic moiety, piroctone and its basic counterpart, ethanolamine that ionizes in solution as per Equation (2):

(2) $$C_{16}H_{30}N_{2}O_{3}\to C_{14}H_{23}NO_{2}+C_{2}H_{7}\mathrm{NO}$$

**FIGURE 3:**
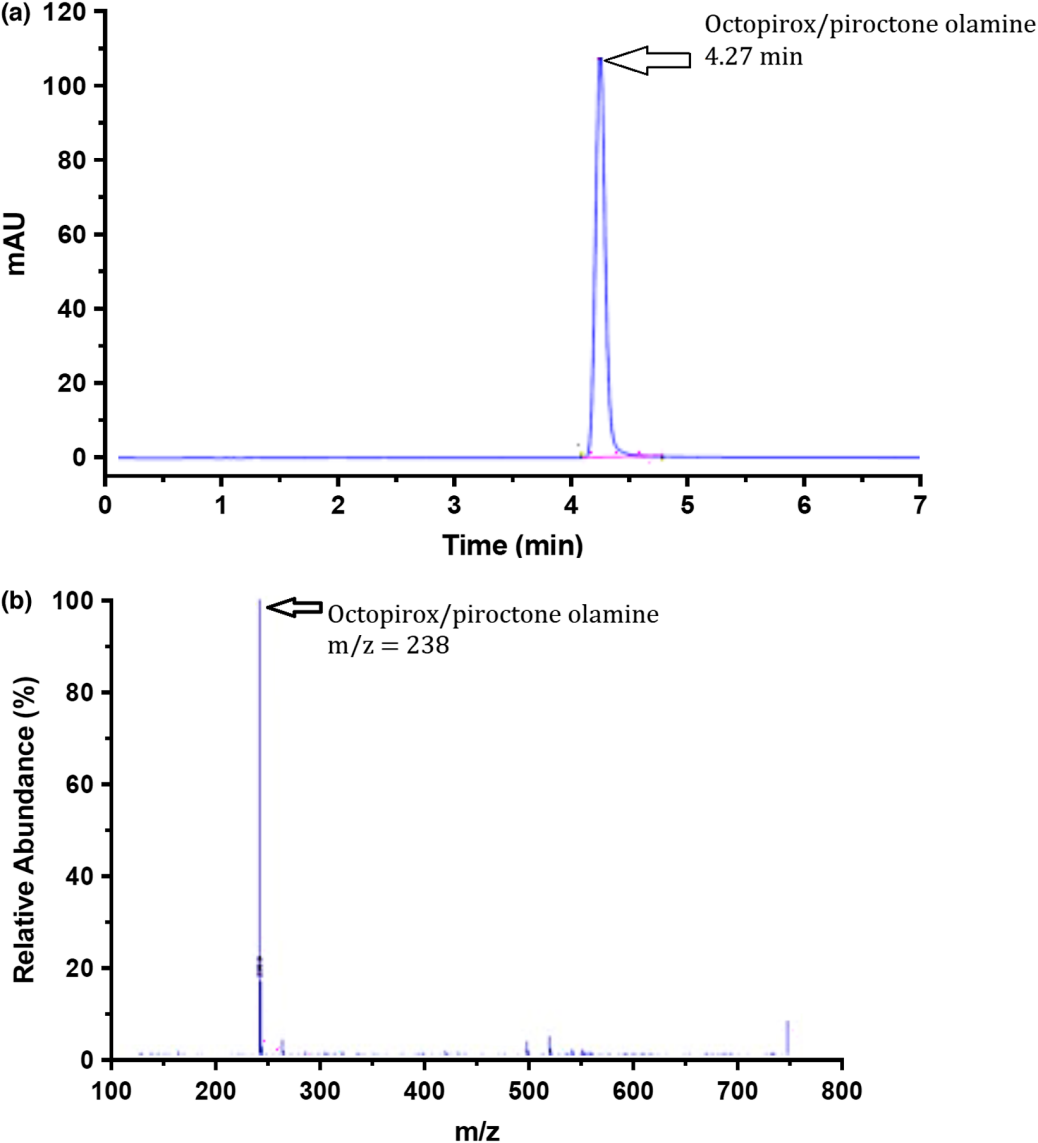
50 μg mL^−1^ standard of OPX in methanol in (a) HPLC chromatogram and (b) LCMS spectrum.

Therefore, for HPLC analysis, only the acidic moiety is detected (Figure 3). Piroctone has a molecular weight of 237 g mol^−1^, adding one proton to form the molecular ion detected by mass spectrometry as shown in Figure 3b. Given the higher polarity of the ethanolamine, it would be expected to elute within the first minutes of the HPLC run.

### Distribution and partition coefficient determination (log *D*_O_ _/PBS_, log *P*_O_ _/W_)

As OPX has two ionisable microspecies at different pH values, log D will be used to describe its octanol/aqueous partition behaviour. As reported in Table 3, OPX showed values of log *D*_(O/PBS)_ > 1 at pH 7.4 (buffered). This means the piroctone ion presents a higher distribution in octanol following Equation (1):

(3) $$C_{14}H_{23}NO_{2}+H_{2}O⇌C_{14}H_{22}N{O_{2}}^{-}+H_{3}O^{+}$$

(4) $$C_{2}H_{7}\mathrm{NO}+H_{2}O⇌C_{2}H_{8}NO^{+}+OH^{-}$$

**TABLE 3:** Experimental values for log *D*_(O/PBS)_ and log *P*_(O/W)_ of OPX.

Media	Log values
PBS	1.84 ± 0.08 (Log *D*_(O/PBS)_)
Water	0.95 ± 0.09 (Log *P*_(O/W)_)

A decrease in the values for log *P*_O/W_ in DI water was also expected. The equilibrium presented in Equation (3) corresponding to the piroctone moiety is dictated by a pK_a_ of 6.87. Equation (4) shows the dissociation of the ethanolamine with a pKa of 9.55. The overall pH of a solution in DI water will be determined by the two equilibria and the magnitude of these constants [12]. Given that the pK_a_ for the ethanolamine is higher, the solution would be expected to be basic. In basic pH environments, the piroctone would be expected to remain mostly unprotonated and negatively ionized. An ionized molecule would be expected to partition more into the aqueous phase, which is observed for OPX as shown in Table 3.

OPX showed low recovery in aqueous solvents during stability studies which was addressed with the addition of a solubilizer. For the evaluation of the log *D*, OPX was initially dissolved in *n*‐octanol and only the portion able to partition into the aqueous phase was detected. Hence, no degradation was observed confirmed by high recovery in both octanol and aqueous phases.

### Solubility and stability studies

**FIGURE 4:**
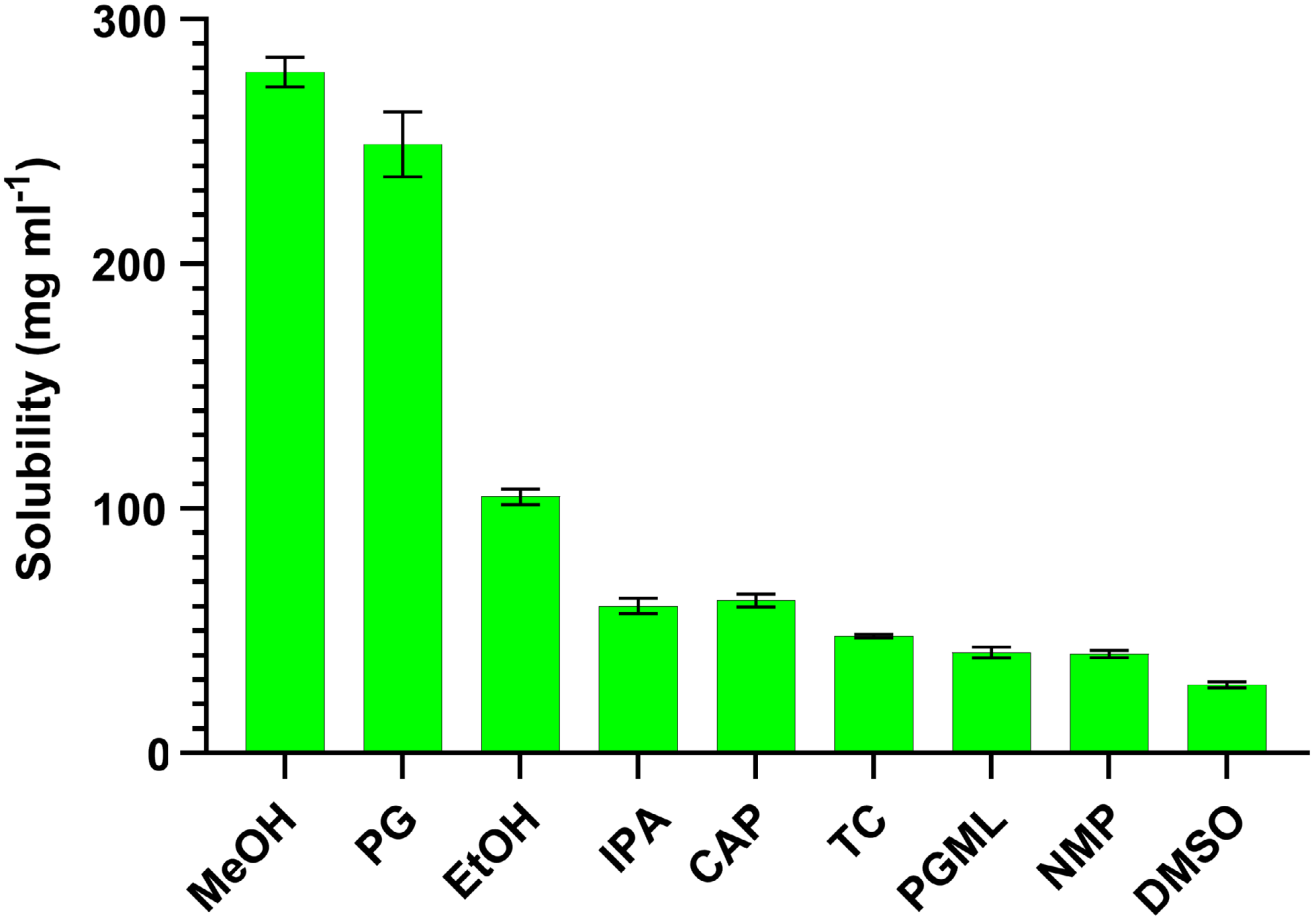
Solubility of OPX in relevant solvents at 32 ± 1°C. *N* = 3, mean ± SD.

Mass balance studies are conducted to allow a full understanding of the fate of the active following application to the skin in a topical formulation. These studies provide information on how much active is left on the skin, how much is deposited in the skin and how much of the active permeates the skin. As shown in Figure 4, the solubility in MeOH was almost double that in EtOH so the former solvent was chosen to conduct mass balance studies. To our knowledge, this has not been reported previously. DMSO was also considered for mass balance studies but was not used because of the low solubility of OPX in this solvent (27.9 ± 1.2 μg mL^−1^). OPX solubility in water is only 7.2 ± 0.5 mg mL^−1^. As reported by Selzer et al. [13], sink conditions would be maintained as long as the concentration of active in the receptor media does not exceed 10% of its solubility. Given that OPX will be applied as 1% (w/v) solutions, it would easily reach 10% of its solubility in PBS, therefore a solubilizer must be added to the receptor media to maintain sink conditions while the integrity of the membrane should be unaffected [14]. Different receptor fluids have been proposed in the literature and Bronaugh [15] discussed the lower absorption values observed when mammalian proteins were added to the receptor media. In their investigation, the use of non‐ionic surfactants was proposed due to the mild effect on the skin and comparatively lower irritation potential. A polyethylene oleyl ether surfactant (Brij® O20) at 5% (w/v) significantly increased the solubility of OPX and would need to be included in the receptor medium to ensure sink conditions during skin permeation studies.

**FIGURE 5:**
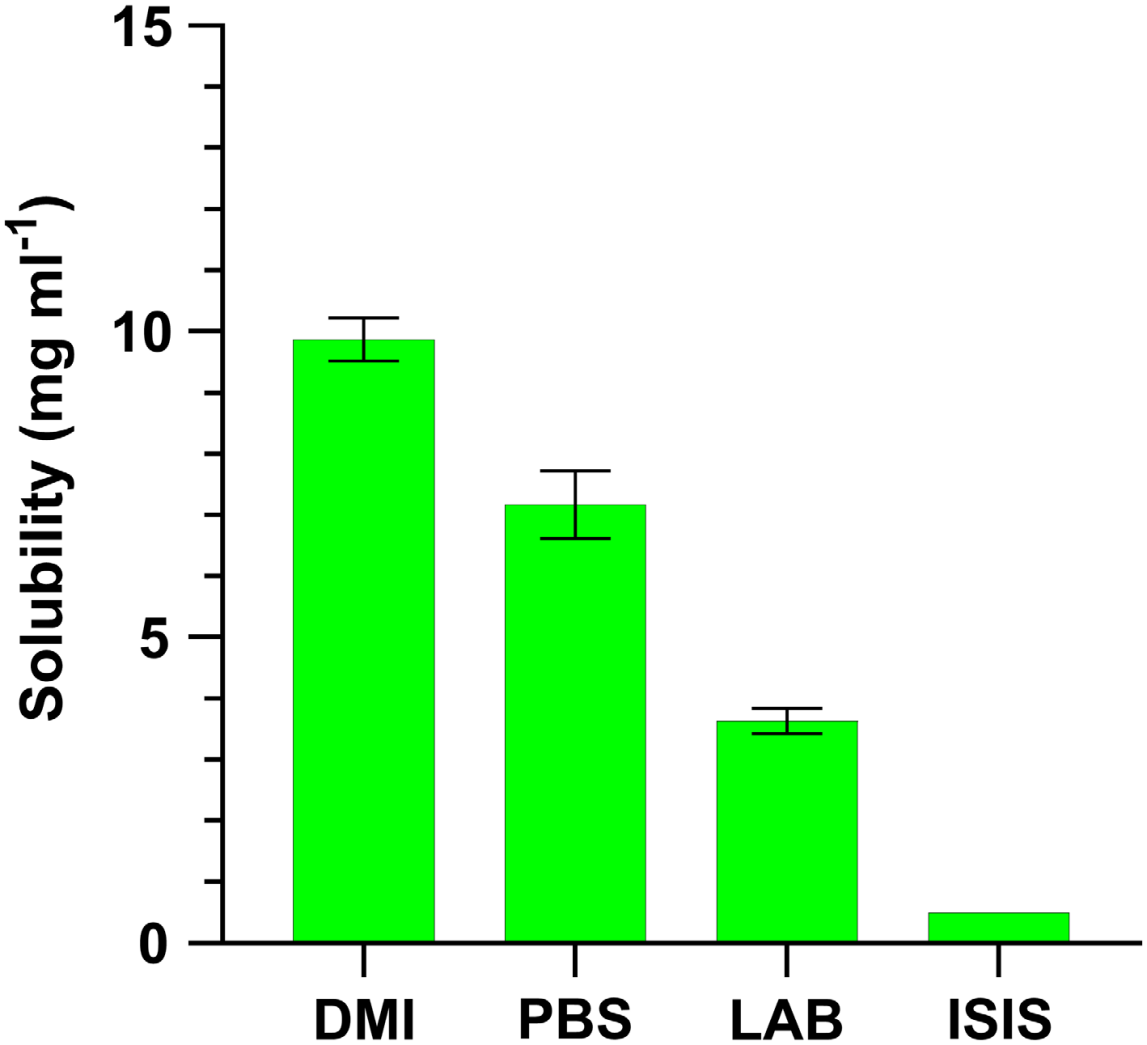
Selection of solvents in which the solubility of OPX is <10 mg mL^−1^ at 32 ± 1°C. *n* = 3, mean ± SD.

OPX showed the lowest solubility in DMI, LAB and ISIS (Figure 5). The effect of saturation has been described as a method for passively promoting penetration [16, 17]. Using systems close to saturation increases the thermodynamic activity of the actives and promotes partitioning into the skin in order to achieve more stable systems, so concentrations lower than 1% in saturated systems might be explored as a strategy to increase OPX skin uptake.

**FIGURE 6:**
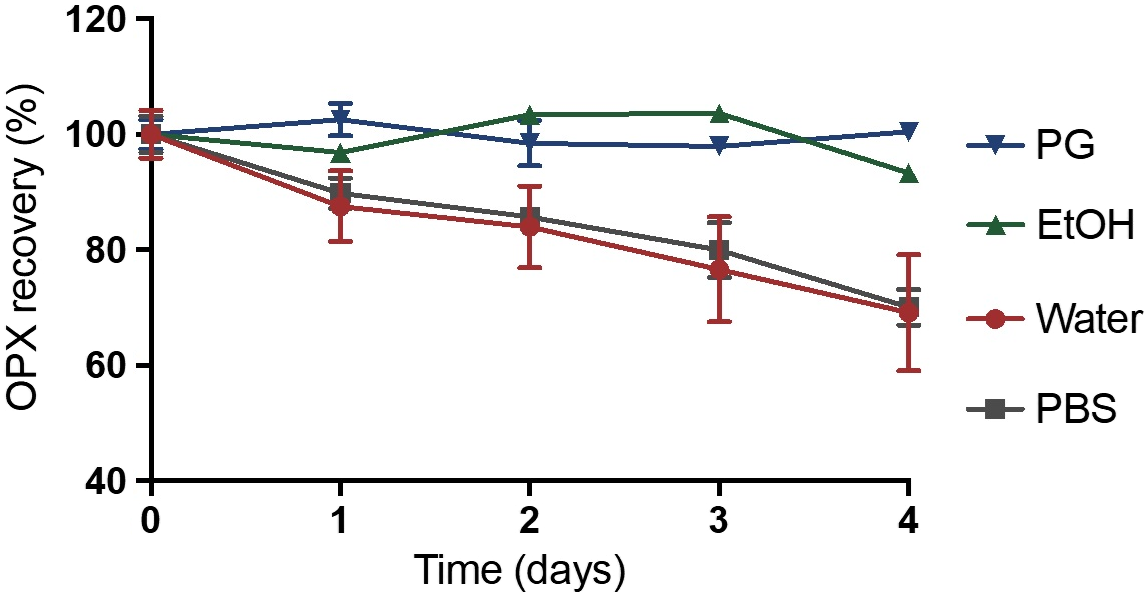
Stability of OPX in selected solvents at 32 ± 1°C, *n* = 3, mean ± SD.

Stability of OPX at 32 ± 1°C in a number of relevant solvents for in vitro studies was studied over 4 days (Figure 6). OPX showed stability in EtOH, PG and the remaining solvents (data not shown for clarity) over 4 days, while a notable decrease in the amounts of OPX was observed in PBS and water. The stability of OPX in water has been reported elsewhere [18], where the degradation was only attributed to light. However, after LCMS analysis, it was confirmed that OPX unlikely to degrade as the only peak present in the mass spectrum was a value of *m*/*Z* = 238, corresponding to the molecular ion of piroctone. It was observed that OPX was poorly soluble in water and PBS, and upon suspension, the consistency of the powder changed to a flaky suspension.

**FIGURE 7:**
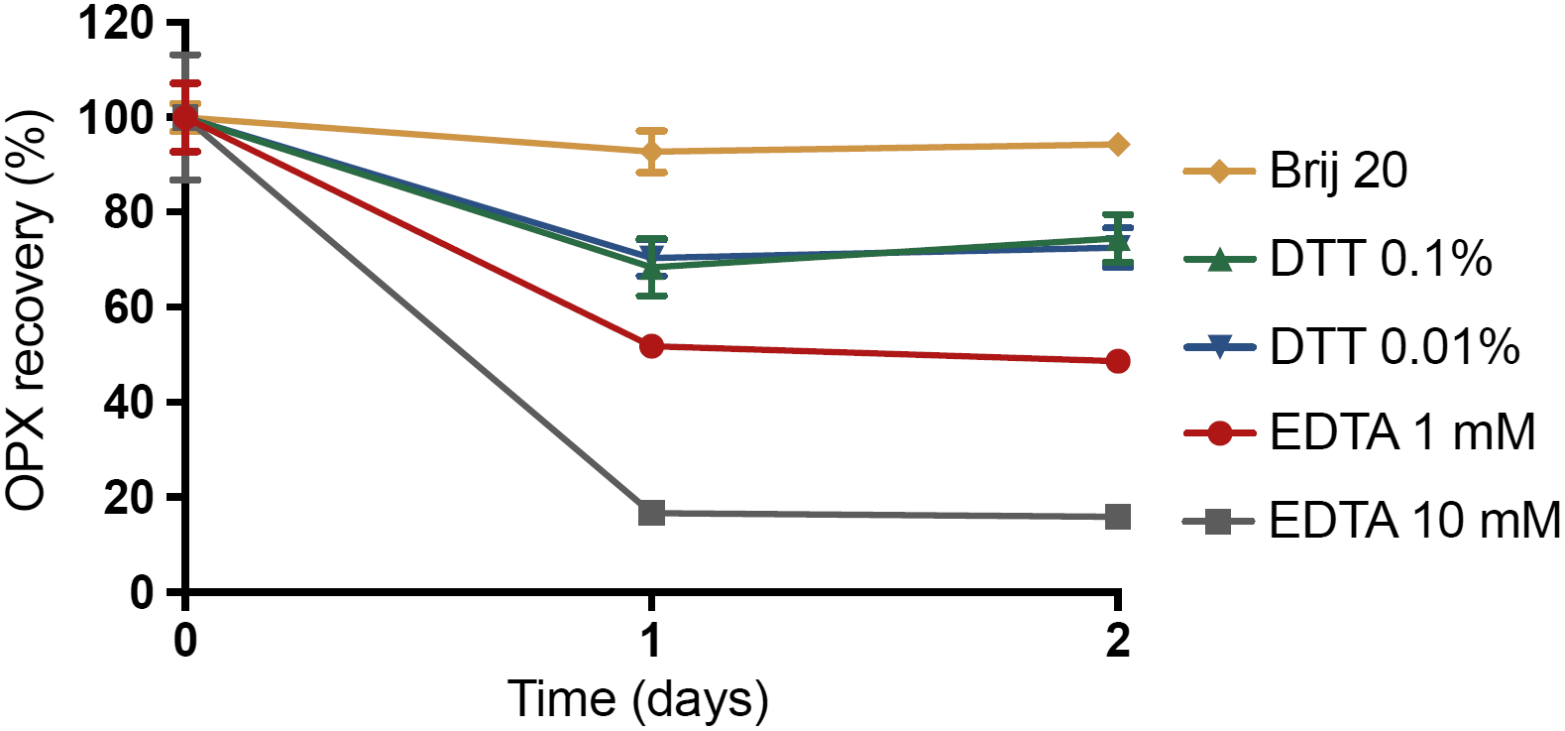
Stability of OPX in a range of preservatives/solubilizers in PBS at 32°C ± 1, *n* = 3, mean ± SD.

A number of stabilizing agents were selected to encompass a range of possible degradation pathways for OPX. EDTA is commonly used as an antimicrobial chelating agent [19]. If there were an interaction between OPX and the salts present in PBS causing precipitation, EDTA could reduce it, but as shown in Figure 7, EDTA did form complexes with piroctone in a dose‐dependent manner, resulting in an even lower recovery. DL‐dithiothreitol (DTT) acts by decreasing the oxidation of molecules [20]. Piroctone presents two groups susceptible of oxidation, where DTT could counteract degradation. As shown in Figure 7, even at higher concentrations, DTT was not able to stabilize OPX.

During solubility studies, 5% Brij® O20 was added to PBS to allow for higher concentrations in the proposed receptor medium and to maintain sink conditions during in vitro studies. When the stability of OPX was investigated in PBS with 5% Brij, a constant concentration could be maintained. 1 mg mL^−1^ solutions were used for stability studies, and only once Brij was added and the solubility increased, the solutions were stable. Therefore, this is likely not a problem of stability but, rather, OPX precipitation during the incubation periods once piroctone and the ethanolamine group were ionized and solvated.

## CONCLUSIONS

The physicochemical properties of OPX were characterized to find suitable excipients able to target the epidermis for topical delivery. An HPLC method for the active ingredients was developed and validated according to ICH guidelines for quantification. Melting point, log *D*, solubility and stability studies were evaluated since these physicochemical properties were not available in the literature. OPX dissociates into two ionized species in solution, piroctone and ethanolamine. The melting point was determined as 132.4°C and a log *D*_o/PBS_ value of 1.8 at physiological pH was confirmed. Additionally, the low water solubility of the molecule had to be addressed for future in vitro penetration studies where sink conditions will be required. The addition of a non‐ionic surfactant was evaluated and Brij® was identified as a good candidate solubilizer for in vitro studies. OPX also showed stability in solution for up to 4 days, which will allow the performance of permeation studies and analytical quantification within a known time frame of stability. Future work will build on the results reported here for the development and evaluation of novel topical formulations of OPX.
